# Etiology and Outcomes of Pediatric Chest Pain in the ED: A Systematic Review

**DOI:** 10.7759/cureus.105526

**Published:** 2026-03-19

**Authors:** Maha Mohamed Sallam Mohamed, Hagar Elhag Mohamed Elamin, Suha Mahmoud, Aya Abdalla, Sara khalel Mohammed, Sarah Othman Hussien Abdaallah, Gihan Hassan Ali Ahmed, Hallah M Suliman

**Affiliations:** 1 Paediatrics, Independent Research, Hull, GBR; 2 Paediatrics, Shaqra General Hospital, Riyadh, SAU; 3 Paediatrics, Independent Research, St. George's, GRD; 4 Pathophysiology, St. George's University, St. George's, GRD; 5 Paediatrics, Alshroq Children Hospital, Benghazi, LBY; 6 Paediatrics and Child Health, United Arab Emirates Ministry of Health and Prevention (MoHAP), Dubai, ARE; 7 Paediatrics, University of Kassala, Kassala, SDN; 8 Paediatrics and Child Health, University Hospital Limerick, Limerick, IRL

**Keywords:** cardiac causes, emergency department, etiology, pediatric chest pain, systematic review

## Abstract

Chest pain is a frequent complaint among children presenting to EDs, often raising concerns about underlying cardiac disease. This systematic review aimed to synthesize available evidence on the etiology, clinical outcomes, and prognosis of pediatric chest pain in ED settings.

A systematic search of PubMed, BMJ Journals, Scopus, IEEE Xplore, and Web of Science was conducted to identify relevant studies. Studies reporting original data on children (<19 years) presenting with chest pain in ED settings were included. Study quality was assessed using the Newcastle-Ottawa Scale. Due to heterogeneity among the studies, a narrative synthesis of the findings was performed.

Six retrospective studies comprising 14,871 patients from five countries met the inclusion criteria. Idiopathic chest pain was the most common etiology, accounting for 24.4%-45.4% of cases. Musculoskeletal (4.7%-33.0%), respiratory (2.9%-17.7%), and psychogenic causes (6.0%-21.6%) were also frequently reported. Cardiac etiology was rare in unselected populations (0.9%-1.5%), although a higher prevalence (7.1%) was observed in ambulance-attended cohorts. Hospitalization rates ranged from 0.3% to 7.2%, and cardiac interventions were required in fewer than 0.3% of patients. Mortality was reported to be below 0.2% across all studies. Five studies were rated as good quality, while one study was rated as fair quality.

Pediatric chest pain in ED settings is predominantly benign, with idiopathic causes being the most common and cardiac pathology being rare. Mortality is exceptionally low, supporting reassurance and targeted evaluation rather than routine extensive diagnostic testing.

## Introduction and background

Chest pain is a frequent and often alarming symptom prompting pediatric visits to the ED. Although chest pain in adults is commonly associated with life-threatening cardiac conditions, its clinical significance in children differs substantially [1]. In the pediatric population, most cases are ultimately attributed to benign and self-limiting causes, yet the presenting complaint often generates considerable anxiety among patients, caregivers, and clinicians [2]. This discrepancy between perceived severity and actual risk frequently leads to extensive diagnostic evaluation, increased healthcare utilization, and variability in management strategies across institutions [3].

The epidemiology of pediatric chest pain in the ED has been explored in multiple regional and single-center studies, which consistently demonstrate that non-cardiac etiologies predominate. Musculoskeletal causes, including costochondritis and chest wall strain, are commonly reported as the leading contributors [4]. Other frequent etiologies include respiratory conditions such as asthma and pneumonia, GI disorders such as gastroesophageal reflux, and psychogenic or anxiety-related causes. True cardiac etiologies, such as myocarditis, pericarditis, arrhythmias, or structural heart disease, are comparatively rare but remain the primary concern during the initial assessment because of their potential severity [5]. The challenge for emergency clinicians lies in accurately identifying the small subset of children at risk for serious cardiac pathology while avoiding unnecessary investigations and hospital admissions for low-risk patients.

Despite numerous individual studies, there remains heterogeneity in the reported prevalence rates of different etiologies, definitions of diagnostic categories, and reported outcomes such as hospitalization rates, the need for cardiac intervention, and mortality [3]. Additionally, variations in study design, geographic location, age distribution, and referral patterns contribute to inconsistencies in the literature [6]. Some studies focus primarily on diagnostic yield, while others evaluate clinical predictors, resource utilization, or follow-up outcomes [7]. As a result, a comprehensive synthesis of the current evidence is essential to clarify the relative distribution of etiologies and to better understand short- and intermediate-term clinical outcomes in children presenting to the ED with chest pain.

Understanding the true etiological spectrum and associated outcomes has important clinical implications. Accurate risk stratification can improve patient safety by facilitating early recognition of serious conditions while also reducing unnecessary testing, radiation exposure, healthcare costs, and ED length of stay [7, 8]. Furthermore, synthesizing contemporary evidence can inform the development of standardized clinical pathways and evidence-based guidelines for evaluation and management in emergency settings.

Therefore, this systematic review aims to comprehensively evaluate the etiology and clinical outcomes of pediatric chest pain in the ED. By synthesizing available data from original studies, this review seeks to quantify the relative frequency of major etiological categories and assess key outcomes, including hospitalization rates, the requirement for cardiac interventions, and mortality. The findings are intended to provide clinicians, researchers, and policymakers with an updated, evidence-based understanding of pediatric chest pain presentations in the ED, ultimately contributing to improved clinical decision-making and optimized patient care.

## Review

Methodology

Study Design and Protocol

This systematic review was conducted and reported in accordance with the Preferred Reporting Items for Systematic Reviews and Meta-Analyses (PRISMA) 2020 statement [9] to ensure methodological transparency, reproducibility, and completeness of reporting. The review process was designed a priori, including clearly defined eligibility criteria, search strategy, study selection process, and methods for data extraction and risk-of-bias assessment.

Eligibility Criteria (PICOS Framework)

The eligibility criteria were defined using the PICOS framework.

Population (P): Studies involving pediatric patients aged 0-18 years presenting with chest pain to EDs were included. Studies focusing exclusively on adult populations were excluded unless pediatric data were reported separately.

Intervention/Exposure (I): The exposure of interest was presentation to the ED with chest pain, regardless of suspected etiology. No specific diagnostic or therapeutic intervention was required for inclusion.

Comparison (C):As this review primarily aimed to describe etiological distribution and outcomes, a comparison group was not mandatory. However, studies comparing different etiologies or risk-stratification strategies within pediatric ED populations were eligible.

Outcomes (O):Studies were required to report at least one of the following outcomes: distribution of etiologies (e.g., musculoskeletal, cardiac, respiratory, GI, psychogenic, idiopathic), hospitalization rates, requirement for cardiac intervention, or mortality.

Study design (S): Original observational studies, including retrospective and prospective cohort studies and cross-sectional studies, were included. Case reports, case series with very small sample sizes, reviews, editorials, conference abstracts without full text, and non-English publications were excluded. To ensure the inclusion of the most recent and clinically relevant evidence, only studies published within the last five years (2021-2025) were considered.

Information Sources and Search Strategy

A comprehensive literature search was performed in the following electronic databases: PubMed, BMJ Journals, Scopus, IEEE Xplore, and Web of Science. The search strategy combined controlled vocabulary terms (e.g., MeSH terms) and free-text keywords related to “pediatric,” “children,” “chest pain,” “emergency department,” “etiology,” and “outcomes.” Boolean operators (AND, OR) were used to refine the search. The reference lists of included studies were also screened manually to identify additional relevant articles. The detailed search strategy for each database is provided in Appendix 1.

Study Selection

All identified records were imported into EndNote X9 for management and duplicate removal. After automatic and manual duplicate screening, titles and abstracts were independently assessed for eligibility. Full texts of potentially relevant studies were retrieved and evaluated against the predefined inclusion and exclusion criteria. Any discrepancies during the selection process were resolved through discussion to reach consensus.

Data Extraction

A standardized data extraction form was developed prior to data collection. Extracted data included study characteristics (country, study design, setting, sample size, age distribution, percentage of male participants, and study period), distribution of etiological categories, hospitalization rates, requirement for cardiac interventions, and mortality rates. Data extraction was performed independently by two reviewers to minimize bias, with discrepancies resolved through discussion and consensus to ensure accuracy and consistency across studies.

Risk of Bias Assessment

The methodological quality and risk of bias of included studies were assessed using the Newcastle-Ottawa Scale (NOS) for observational studies [10]. This tool evaluates studies based on the selection of participants, comparability of study groups, and assessment of outcomes. Studies were categorized as low, moderate, or high risk of bias according to their NOS scores.

Data Synthesis

A qualitative synthesis of findings was conducted. Although quantitative pooling through meta-analysis was initially considered, it was not performed because of substantial clinical and methodological heterogeneity among the included studies. Specifically, there were significant variations in age ranges, diagnostic classifications, definitions of etiological categories, outcome-reporting formats, healthcare settings, and follow-up durations. Furthermore, inconsistent reporting of outcome measures and the lack of standardized denominators limited the feasibility of reliable statistical pooling. Conducting a meta-analysis under these conditions could have produced misleading summary estimates and compromised the validity of the conclusions. Therefore, a structured narrative synthesis was deemed more appropriate to accurately reflect the diversity of the available evidence and to provide a clinically meaningful interpretation of the findings.

Results

Study Selection

**Figure 1:**
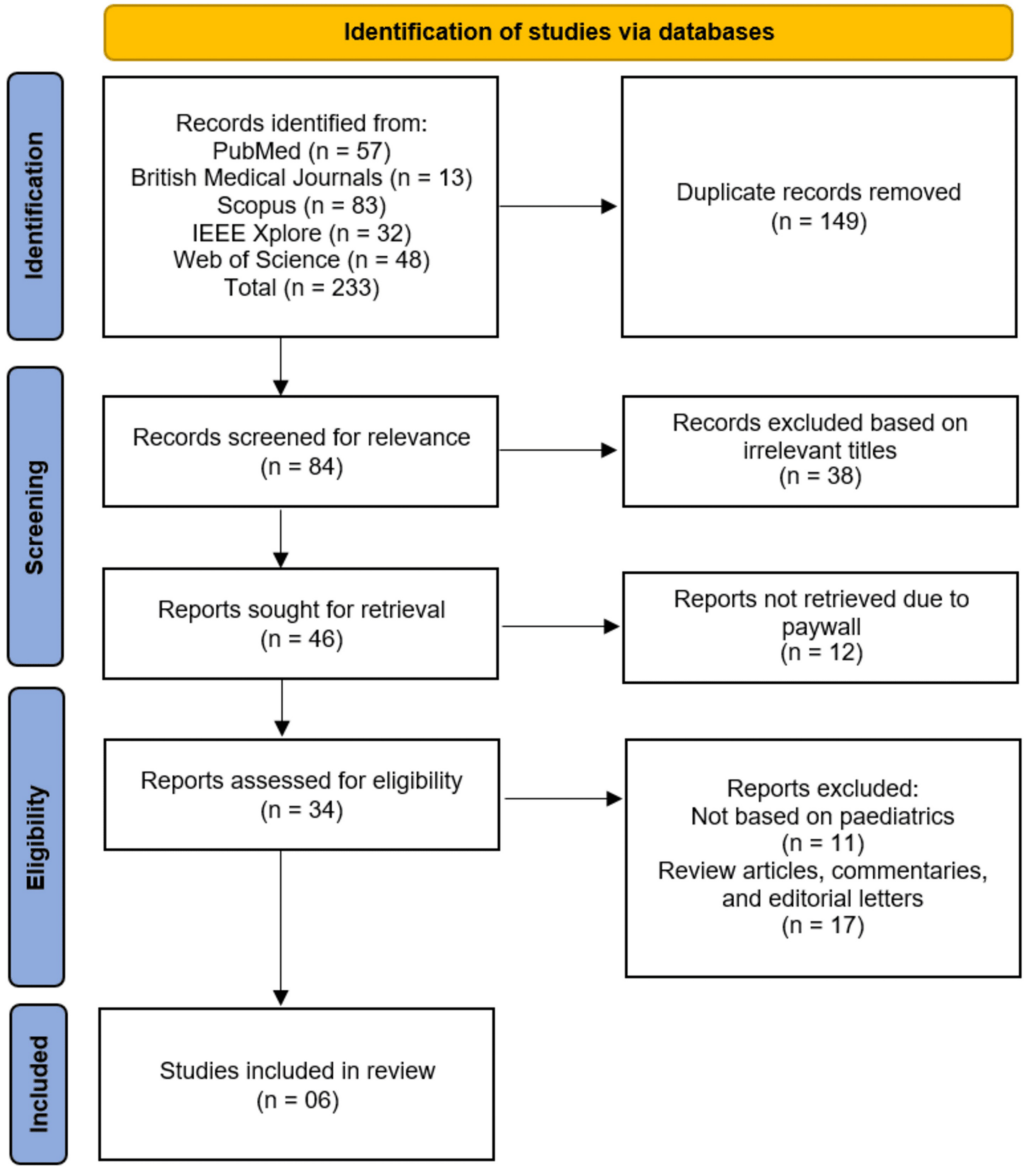
Study selection process illustrated in the PRISMA flowchart. PRISMA: Preferred Reporting Items for Systematic Reviews and Meta-Analyses.

The study selection process followed the PRISMA guidelines, as illustrated in Figure 1. A comprehensive literature search across five electronic databases, PubMed, BMJ Journals, Scopus, IEEE Xplore, and Web of Science, yielded a total of 233 records. After removing 149 duplicate records, 84 unique studies remained for initial screening. Following title and abstract screening, 38 records were excluded due to irrelevant titles, leaving 46 reports sought for retrieval. Of these, 12 reports could not be retrieved due to paywall restrictions. The remaining 34 full-text reports were assessed for eligibility against the predefined inclusion criteria. During this phase, 11 reports were excluded because they were not based on pediatric populations, and a further 17 reports were excluded because they were review articles, commentaries, or editorial letters. Ultimately, six studies met all inclusion criteria and were included in this systematic review [11-16].

Studies Characteristics

A total of six retrospective studies met the inclusion criteria for this systematic review, encompassing 14,871 pediatric patients who presented to EDs with chest pain [11-16]. The included studies were conducted across diverse geographical regions, including Australia [11], the United States [12, 14], Saudi Arabia [13], Portugal [15], and Italy [16]. All studies employed retrospective cohort or observational designs, with data collection periods ranging from 2015 to 2022. The characteristics of each included study are summarized in Table 1.

**Table 1:** Characteristics of included studies evaluating pediatric chest pain in the ED. CHUSJ: Centro Hospitalar Universitário de São João; NR: Not reported; PICU: Pediatric intensive care unit.

Author (Year)	Country	Study Design	Setting	Sample Size (n)	Age Range / Mean Age	% Male	Study Period	Inclusion Criteria	Key Objective
Okyere D et al. [11] (2025)	Australia	Retrospective cohort	Statewide EMS and linked ED/hospital data	4,277 cases	<18 years; median age 14 years	41%	2015-2019	EMS-attended non-traumatic chest pain (<18 years)	Incidence, etiology, and short-term outcomes
Atmanli A et al. [12] (2024)	USA	Retrospective	Pediatric ED, Children’s Medical Center (Dallas and Plano, TX)	10,721	2-17 years (mean 12 years)	47.60%	2018-2022	Chest pain, no prior cardiac disease	To evaluate the causes, testing, and outcomes of pediatric chest pain before versus during COVID-19
Alnaim AA et al. [13] (2023)	Saudi Arabia	Retrospective cohort	Pediatric ED	310	1-13 years; mean age 9.1 ± 2.7 years	18.40%	2017-2022	Non-traumatic chest pain, age 1-14 years	To describe the etiology, evaluation, and outcomes of pediatric chest pain
Babbitt CJ et al. [14] (2022)	USA	Retrospective cohort	PICU (following ED presentation)	36 (myopericarditis), 22 (myocarditis)	Median age 16.2 years	97%	9 years	Discharge diagnosis of myopericarditis or myocarditis	To differentiate myopericarditis from myocarditis in the ED setting
Pissarra R et al. [15] (2022)	Portugal	Retrospective	CHUSJ Pediatric ED, Porto	798	1-17 years (mean 13 years)	46%	2018	All ED visits for chest pain	To describe presentation, causes, investigations, and outcomes
Brancato F et al. [16] (2021)	Italy	Retrospective observational	Pediatric ED	107	0-19 years	NR	NR	Children admitted for chest pain	To evaluate the usefulness of plasma troponin level determination in the initial differential diagnosis of pediatric chest pain

The sample sizes varied considerably across studies, ranging from 58 patients in a focused investigation of myopericarditis and myocarditis [14] to 10,721 patients in a large multicenter study examining diagnostic testing patterns before and during the COVID-19 pandemic [12]. The age ranges across studies spanned from infancy to 19 years, with mean ages typically falling within the early to mid-adolescent period. The proportion of male patients ranged from 18.4% [13] to 97% [14], with the latter representing a selected population of patients with myopericarditis.

Etiology of Pediatric Chest Pain

The etiological distribution of pediatric chest pain varied substantially across the included studies, reflecting differences in study populations, settings, and classification systems, as detailed in Table 2. Idiopathic chest pain emerged as the most common diagnostic category in studies that reported this outcome, accounting for 42.2% of cases in the Australian cohort study by Okyere D et al. [11] and 45.4% in the Italian study by Brancato F et al. [16]. Similarly, Pissarra R et al. reported that 24.4% of cases in their Portuguese cohort remained unexplained [15].

**Table 2:** Etiology and clinical outcomes of pediatric chest pain in the ED.

Author (Year)	Total Patients	Musculoskeletal (%)	Cardiac (%)	Respiratory (%)	Gastrointestinal (%)	Psychogenic (%)	Idiopathic (%)	Hospitalization Rate (%)	Cardiac Intervention Required (%)	Mortality (%)
Okyere D et al. [11] (2025)	4,277	4.70%	7.10%	17.70%	2.60%	6.00%	42.20%	7.20%	NR	<0.2%
Atmanli A et al. [12] (2024)	10,721	NR	0.90%	NR	NR	NR	NR	NR	NR	NR
Alnaim AA et al. [13] (2023)	310	NR	1.50%	2.90%	NR	NR	NR	0.30%	0.30%	0%
Babbitt CJ et al. [14] (2022)	58	NR	100%	NR	NR	NR	NR	100%	NR	0%
Pissarra R et al. [15] (2022)	798	33.00%	1.10%	12.80%	5.30%	21.60%	24.40%	2.80%	0.25%	0%
Brancato F et al. [16] (2021)	107	NR	5.60%	NR	NR	NR	45.40%	NR	NR	0%

Musculoskeletal causes represented a significant proportion of identified etiologies, ranging from 4.7% in the prehospital setting [11] to 33% in the Portuguese ED study [15]. The higher proportion reported by Pissarra R et al. likely reflects the inclusion of all ambulatory ED presentations, whereas the study by Okyere D et al. captured only ambulance-attended cases, potentially representing a more acute or severe cohort [11, 15].

Respiratory conditions were consistently identified as important contributors to pediatric chest pain, with prevalence rates of 17.7% [11], 12.8% [15], and 2.9% [13]. GI etiologies were reported in two studies, accounting for 2.6% [11] and 5.3% [15] of cases, respectively. Psychogenic causes were documented in 6.0% of patients in the Australian study [11] and 21.6% of patients in the Portuguese cohort [15], suggesting that psychological factors may be underrecognized in some clinical settings.

Cardiac Etiologies and Outcomes

The prevalence of cardiac pathology as the underlying cause of pediatric chest pain was consistently low across the included studies. Atmanli A et al. reported the lowest cardiac etiology rate, at 0.9%, in their large multicenter study from Texas [12], followed by Pissarra R et al. at 1.1% [15], Alnaim AA et al. at 1.5% [13], and Brancato F et al. at 5.6% [16]. The study by Okyere D et al. reported a cardiac etiology rate of 7.1%; however, this included patients attended by ambulance services and may reflect a population with higher acuity [11]. Notably, the study by Babbitt CJ et al. focused exclusively on patients with myopericarditis (n=36) and myocarditis (n=22), representing a selected population with confirmed cardiac pathology [14].

Hospitalization and Interventions

Hospitalization rates varied considerably based on the underlying etiology and clinical context. The highest hospitalization rate (100%) was observed in patients with myopericarditis and myocarditis [14]. Among unselected pediatric chest pain populations, hospitalization rates ranged from 0.3% [13] to 7.2% [11]. Cardiac intervention was required in only 0.25% of patients in the Portuguese study [15] and 0.3% of patients in the Saudi Arabian cohort [13].

Mortality and Prognosis

Mortality was an exceedingly rare outcome across all included studies. Okyere D et al. reported a mortality rate of less than 0.2% among ambulance-attended pediatric chest pain patients [11], while the remaining five studies reported no deaths during their respective follow-up periods [12-16]. This consistently low mortality rate across diverse healthcare settings and populations reinforces the generally benign nature of pediatric chest pain presentations to EDs.

Risk of Bias Assessment Results

The methodological quality of the six included retrospective cohort studies was assessed using the NOS, as presented in Table 3. Five studies were rated as good quality, with scores ranging from 6 to 9 stars [11-15], while one study was rated as fair quality, with a score of 4 stars [16]. The studies by Okyere D et al. [11] and Atmanli A et al. [12] achieved the maximum score of 9 stars, demonstrating robust methodological quality through the use of large, representative samples, clearly defined inclusion criteria, and objective outcome ascertainment via linked data systems or electronic health records. Pissarra R et al. [15] also demonstrated good quality with a score of 8 stars, although limited adjustment for confounding factors was noted. Alnaim AA et al. [13] and Babbitt CJ et al. [14] each received 6 stars, reflecting good overall quality but with limitations, including modest sample sizes, single-center designs, and lack of long-term follow-up data. Brancato F et al. [16] received the lowest score of 4 stars and was categorized as fair quality due to a restricted study population of admitted children, absence of confounder adjustment, and unclear specification of the study period. Overall, the included studies demonstrated moderate to high methodological quality, although heterogeneity in design and variable control for confounding should be considered when interpreting the pooled findings.

**Table 3:** Risk of bias assessment using the Newcastle-Ottawa Scale (NOS) for cohort studies.

Author (Year)	Selection (Max 4)	Comparability (Max 2)	Outcome (Max 3)	Total Score	Quality Rating
Okyere D et al. [11] (2025)	★★★★	★★	★★★	9/9	Good
Atmanli A et al. [12] (2024)	★★★★	★★	★★★	9/9	Good
Alnaim AA et al. [13] (2023)	★★★	★	★★	6/9	Good
Babbitt CJ et al. [14] (2022)	★★★	★	★★	6/9	Good
Pissarra R et al. [15] (2022)	★★★	★★	★★★	8/9	Good
Brancato F et al. [16] (2021)	★★	☆	★★	4/9	Fair

Discussion

This systematic review synthesized evidence from six retrospective studies including 14,871 pediatric patients presenting to EDs with chest pain. Overall, the findings indicate that pediatric chest pain is predominantly benign. Idiopathic causes were the most frequently reported, cardiac etiologies were rare in unselected populations, and mortality was exceedingly uncommon. These findings reinforce the generally favorable prognosis of pediatric chest pain and highlight the importance of balanced clinical evaluation that avoids unnecessary testing while ensuring appropriate identification of serious conditions.

Idiopathic chest pain was the most common diagnostic category, accounting for 24.4% to 45.4% of cases across studies reporting this outcome [11, 15, 16]. These results are consistent with previous literature showing that a large proportion of pediatric chest pain remains unexplained despite evaluation. Earlier work has reported similar proportions, with idiopathic causes accounting for approximately 20%-45% of cases depending on the clinical setting and diagnostic approach [17]. Likewise, prospective ED data have demonstrated that many children with chest pain do not receive a specific diagnosis following clinical assessment [18]. The persistence of this pattern across studies suggests that idiopathic chest pain represents a common clinical entity in pediatric populations. However, some cases labeled as idiopathic may reflect limitations of routine ED assessment. For example, the presence of psychogenic etiologies in several studies [11, 15] suggests that psychosocial factors may contribute to a proportion of cases classified as nonspecific.

Musculoskeletal causes were another major contributor to pediatric chest pain, accounting for up to one-third of cases in some cohorts [15]. These findings are consistent with previous systematic reviews reporting musculoskeletal conditions as the most common identifiable cause in children with chest pain [19]. Variation in reported prevalence likely reflects differences in study populations and healthcare utilization patterns. For instance, musculoskeletal causes were less frequent in ambulance-attended cohorts [11], possibly because children with mild or subacute symptoms are less likely to activate EMS. Most musculoskeletal etiologies in children, including costochondritis and precordial catch syndrome, are benign and self-limited, emphasizing the importance of accurate clinical recognition to avoid unnecessary investigations.

Respiratory conditions also represented a notable proportion of pediatric chest pain presentations, with prevalence rates varying across studies [11, 15]. Previous research has similarly identified respiratory illnesses, including asthma and pneumonia, as common contributors to chest pain in children [20]. The mechanisms linking respiratory disease to chest pain may include pleural irritation, coughing-related musculoskeletal strain, or referred diaphragmatic pain. Differences in reported prevalence across studies may reflect variations in study populations, seasonal patterns of respiratory illness, or diagnostic classification methods [13].

One of the most clinically important findings of this review is the consistently low prevalence of cardiac pathology. Cardiac causes were reported in approximately 0.9%-1.5% of unselected pediatric chest pain populations [12, 13, 15], with slightly higher rates observed in specific cohorts [16]. These estimates closely align with prior large-scale investigations demonstrating that cardiac disease accounts for only a small proportion of pediatric chest pain cases [21]. A higher prevalence was observed in ambulance-attended cohorts [11], likely reflecting the inclusion of patients with more severe or concerning symptoms. Overall, these findings reinforce the widely recognized principle that cardiac disease is an uncommon cause of chest pain in children presenting to EDs.

The prognosis of pediatric chest pain was also highly favorable. Mortality was extremely rare, with most studies reporting no deaths and only one study documenting mortality below 0.2% [11-16]. Long-term follow-up studies similarly demonstrate excellent outcomes, with very low rates of subsequent cardiac disease among children evaluated for chest pain [22]. The absence of mortality in most cohorts likely reflects both the low prevalence of serious pathology and the effectiveness of current triage and diagnostic practices in emergency care settings.

Hospitalization rates varied across studies, ranging from 0.3% to 7.2% [11, 13], with higher rates observed in populations presenting via ambulance or with more severe clinical features. However, the need for cardiac intervention was extremely rare, occurring in fewer than 0.3% of patients [13, 15]. These findings suggest that most pediatric chest pain presentations can be managed safely without extensive inpatient evaluation. Consequently, structured clinical assessment strategies may help optimize resource utilization while maintaining patient safety.

Quality assessment indicated generally strong methodological quality among the included studies, with five rated as good quality and one as fair quality [11-16]. The higher-quality studies typically included large representative samples and clearly defined outcome measures, while the fair-quality study was limited by its restricted population and methodological constraints [16]. Although these limitations should be considered when interpreting the results, the overall consistency of findings across studies strengthens the reliability of the conclusions.

The included studies were conducted in several high-income countries, including Australia, the United States, Saudi Arabia, Portugal, and Italy. The convergence of findings across these diverse healthcare systems suggests a broadly consistent epidemiological pattern of pediatric chest pain. However, evidence from low- and middle-income countries remains limited. Differences in disease burden, including a higher prevalence of infectious diseases or rheumatic heart disease, may influence etiological patterns in these settings [23]. Future research should therefore include more geographically diverse populations to improve global generalizability.

Several clinical implications emerge from these findings. The consistently low prevalence of cardiac pathology indicates that routine advanced cardiac testing for all children with chest pain is unlikely to be necessary or cost-effective [23]. Instead, clinical evaluation should prioritize careful history-taking and physical examination to identify high-risk features such as exertional symptoms, syncope, abnormal examination findings, or a family history of sudden cardiac death [24]. In addition, the high proportion of idiopathic and psychogenic causes highlights the potential value of psychosocial assessment, particularly among adolescents. Importantly, the overall excellent prognosis supports reassurance as a key component of management, which may reduce anxiety for patients and families while limiting unnecessary healthcare utilization [25].

Limitations

This systematic review has several limitations that should be considered when interpreting the findings. First, all six included studies employed retrospective designs, which are inherently susceptible to selection bias, information bias, and confounding. Retrospective studies rely on the accuracy and completeness of medical records, which may vary across institutions and over time. Second, there was substantial heterogeneity across studies in terms of inclusion criteria, outcome definitions, and classification systems for etiological categories. For example, some studies included only ambulance-attended patients [11], while others included all ED presentations [12, 15], and the definitions of cardiac, respiratory, and psychogenic causes were not standardized. This heterogeneity precluded meta-analysis and limits the precision of the overall estimates. Third, the majority of studies were conducted in single centers, which may limit the generalizability of the findings to other healthcare settings or populations. Fourth, follow-up periods were generally short, ranging from the index ED visit to hospital discharge, with limited data on long-term outcomes or recurrence rates. Fifth, the risk-of-bias assessment revealed variable methodological quality across studies, with one study rated as fair quality [16], introducing the potential for biased estimates. Sixth, the proportion of male participants varied substantially across the included studies, particularly in those examining specific cardiac diagnoses. Although this variation likely reflects differences in study populations rather than methodological bias, sex distribution could potentially influence the observed etiological patterns of pediatric chest pain, as certain cardiovascular or musculoskeletal conditions may show sex-related differences in prevalence. Seventh, publication bias cannot be excluded, as studies with positive findings or unusual cardiac pathology may be more likely to be published than studies confirming the benign nature of chest pain. Eighth, the exclusion of non-English-language studies and the inability to retrieve 12 reports due to paywall restrictions may have introduced selection bias. Finally, the absence of studies from low- and middle-income countries limits the global applicability of our findings, as the epidemiology of pediatric chest pain may differ in settings with different disease burdens and healthcare resources.

## Conclusions

Pediatric chest pain presenting to emergency departments is predominantly benign, with idiopathic causes representing the most common diagnostic category, cardiac pathology accounting for less than 2% of unselected cases, and mortality being an exceedingly rare outcome. Musculoskeletal and respiratory etiologies constitute the most frequent identifiable causes, while psychogenic factors are increasingly recognized, particularly among adolescents. The excellent prognosis and low intervention rates support the development of evidence-based clinical pathways that prioritize targeted evaluation based on specific risk factors rather than routine extensive diagnostic testing. Future research should prioritize prospective multicenter studies with standardized outcome definitions, longer follow-up periods, and the inclusion of diverse geographical and economic settings to enhance the generalizability of the findings. Additionally, studies examining the cost-effectiveness of different diagnostic strategies and the impact of psychosocial interventions on long-term outcomes would further inform clinical practice and healthcare policy.

## Appendices

Appendix 1

**Table 4:** Search strategy for each database.

Database	Search Strategy
PubMed	("Chest Pain"[MeSH] OR "chest pain" OR "thoracic pain") AND ("Child"[MeSH] OR "Adolescent"[MeSH] OR pediatric OR paediatric OR child OR adolescen* OR teen*) AND ("Emergency Service, Hospital"[MeSH] OR "Emergency Department" OR "Emergency Room" OR ED OR emergency care)
BMJ Journals	("chest pain" OR "thoracic pain") AND (pediatric OR paediatric OR child OR adolescen* OR teen*) AND ("emergency department" OR "emergency room" OR "emergency care")
Scopus	TITLE-ABS-KEY ("chest pain" OR "thoracic pain") AND TITLE-ABS-KEY (pediatric OR paediatric OR child OR adolescen* OR teen*) AND TITLE-ABS-KEY ("emergency department" OR "emergency room" OR "emergency care" OR ED)
IEEE Xplore	("chest pain" OR "thoracic pain") AND (pediatric OR paediatric OR child OR adolescen* OR teen*) AND ("emergency department" OR "emergency care")
Web of Science	TS=("chest pain" OR "thoracic pain") AND TS=(pediatric OR paediatric OR child OR adolescen* OR teen*) AND TS=("emergency department" OR "emergency room" OR "emergency care")
